# Synthesis of Silver Nanoparticles by Chemical Vapor Deposition Method and Its Application in Laser Desorption/Ionization Techniques

**DOI:** 10.3390/nano15130973

**Published:** 2025-06-23

**Authors:** Kinga Robotnik, Tomasz Zieliński, Justyna Walczak-Skierska, Ewelina Sibińska, Paulina Rudzik, Piotr Piszczek, Aleksandra Radtke, Paweł Piotr Pomastowski

**Affiliations:** 1 Centre for Modern Interdisciplinary Technologies, Nicolaus Copernicus University in Toruń, Wileńska 4 Str., 87-100 Toruń, Polandwalczak-skierska@umk.pl (J.W.-S.);; 2 Energy Efficiency Project Coordination Team, ORLEN S.A., Chemików 7 Str., 09-411 Płock, Poland; t.zielinski@orlen.pl; 3 Department of Inorganic and Coordination Chemistry, Faculty of Chemistry, Nicolaus Copernicus University in Toruń, Gagarina 7 Str., 87-100 Toruń, Poland; 307387@stud.umk.pl (P.R.); piszczek@umk.pl (P.P.); aradtke@umk.pl (A.R.)

**Keywords:** silver nanoparticles, CVD, LDI-MS, NALDI, MALDI

## Abstract

Laser desorption/ionization techniques, such as matrix-assisted laser desorption/ionization (MALDI) and surface-assisted laser desorption/ionization (SALDI), are the basis of modern mass spectrometry, enabling the analysis of a wide range of chemical compounds, from small molecules to biopolymers. MALDI uses organic matrices to support ionization, while SALDI relies on inorganic surfaces or nanomaterials, which reduce background and improve measurement sensitivity. This review focuses on the potential of using silver nanoparticles (AgNPs) in LDI-MS, with particular emphasis on their synthesis from the gas phase (CVD, Chemical Vapor Deposition). The key role of nanostructures in increasing ionization efficiency and analytical selectivity is emphasized. The CVD technique enables precise control over the morphology, size, and distribution of nanoparticles, which translates into better repeatability and sensitivity of nanostructure-assisted laser desorption/ionization mass spectrometry (NALDI-MS) measurements. The latest achievements in this field are presented, as well as potential applications of CVD-produced AgNPs in analytical chemistry, environmental analysis, and the petrochemical industry.

## 1. Introduction

The development of electronics caused a necessity to develop materials with properties that could satisfy the rising challenges and expectations of the 20th century. Researchers discovered that coating objects with thin layers of oxides or metals could completely modify their chemical characteristics. This discovery caused the development of numerous techniques and methods to create nanomaterials with highly specific properties. Among these, plasmonic materials demonstrate a remarkable ability to interact with various forms of radiation (UV, VIS, IR), which improve modern analytical technologies such as laser desorption/ionization mass spectrometry [1,2,3].

Laser desorption/ionization mass spectrometry (LDI-MS) techniques, including matrix-assisted laser desorption/ionization (MALDI-MS) and surface-assisted laser desorption/ionization (SALDI-MS), revolutionized mass spectrometric analysis. MALDI-MS uses organic matrices to co-crystallize with analytes. These matrices introduce background noise and complicate spectra. To overcome this limitations in SALDI, ionization and desorption are enhanced by various surfaces and their specific structures. A specific variant, nanostructured-assisted laser desorption/ionization, uses nanostructured materials to enhance sensitivity and selectivity [4]. Special attention is given to metal nanoparticles (particularly silver), due to their outstanding electrical conductivity and chemical stability and the relative ease with which they can be synthesized [5]. Achieving uniform and reproducible nanostructures relies heavily on precise control over the synthesis process, particularly in terms of composition, structural arrangement, and morphology of the nanomaterials.

This review focused on the application of nanostructure-assisted LDI techniques in the analysis of low-molecular-weight compounds, with particular emphasis on the use of silver nanoparticles as functional substrates. It presents and compares various synthesis methods for nanostructures, highlighting the importance of selecting appropriate fabrication techniques to ensure reproducibility, sensitivity, and analytical performance. Among the methods discussed, chemical vapor deposition (CVD) was identified as a particularly promising approach due to its ability to precisely control nanoparticle growth and its high reproducibility. The insights provided may support further optimization of substrate design for NALDI applications and guide researchers in choosing effective synthesis strategies for silver-based nanomaterials.

## 2. Organic and Nanomaterial Matrix LDI Platforms

MALDI is an ionization technique most commonly used in connection with a Time-of-Flight (TOF) analyzer. This technique uses matrix—organic acids that absorb UV radiation well and sublimate easily. The matrix used in MALDI includes compounds such as alpha-cyano-4-hydroxycinnamic acid (HCCA) and 2,5-dihydroxybenzoic acid (DHB). There are several methods of applying the analyte onto the MALDI plate, but two of them are more employed. The first involves mixing the analyte with the matrix and placing the resulting mixture directly onto the MALDI plate. The second method includes placing the analyte solution onto the plate, allowing it to dry, and overlaying it with a matrix layer [6].

**Figure 1:**
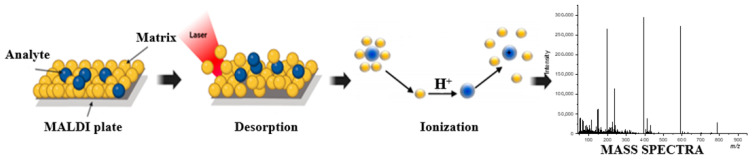
Desorption and ionization processes in matrix-assisted laser desorption/ionization (MALDI).

The matrix absorbs energy from the laser. After desorption, the matrix delivers large amounts of the ions needed to ionize the analyte (see Figure 1). A fundamental necessity for the ionization process in MALDI is the matrix’s ability to absorb laser energy. Optimal MALDI-MS performance is achieved when the laser wavelength (mostly 337 and 355 nm) matches the high optical absorption band of the matrix (e.g., CHCA and DHB λ_max_ occurs at 337 and 353 nm) in the solid phase [7]. Photoabsorption ionization proceeds through a two-step process: first, matrix ionization and, afterwards, ion–molecule reactions producing analyte ions. A Time-of-Flight analyzer used in MALDI-TOF measures their transit time, which is the time that elapses from the moment of ionization of particles in the sample to the moment of their detection by the detector. This time is directly related to the mass-to-charge ratio (*m*/*z*) of the ions because lighter ions move faster than heavier ones under the influence of an electric field. To increase the accuracy and resolution of the measurement, a delayed extraction technique is used, which consists of briefly delaying the application of the extraction voltage after ionization. This delay allows ions with different initial kinetic energies to spatially separate before being accelerated. As a result, ions with the same *m*/*z* ratio, regardless of their initial velocities, can reach the detector at the same time [8,9,10].

The function of the laser it to gently heat the surface, allowing some molecules to evaporate smoothly. This process is usually referred to as desorption, but, in practice, it is difficult to clearly separate it from the ablation phenomenon because both can co-occur depending on the irradiation conditions [11]. The duration and energy of the radiation pulse significantly influence the quality and composition of the mass spectrum [12]. MALDI molecular dynamics simulations have shown that long, low-energy laser pulses (ns, ~10mJ/cm²) promote mild desorption but may be insufficient for efficient material release. In contrast, short, high-energy pulses (ps/fs, hundreds of mJ/cm²) lead to strong ablation; the material undergoes massive disintegration, forming cold fragments and a dense plasma cloud, which lowers the resolution and makes ion collection difficult. This highlights the need for the precise selection of laser parameters to ensure effective desorption without damaging the sample [13,14].

The ionization mechanism in MALDI remains ambiguous and involves several complementary physicochemical models. Classical approaches include photochemical ionization (PI) and cluster ionization (CI). In the PI model proposed by Ehring et al., analyte ions are formed in the gas phase as a result of an ion–molecule reaction, where protonation or deprotonation occurs through collisions with matrix ions. This process is supported by multiphoton absorption and efficient energy storage in the analyte–matrix system. In the CI model, it is assumed that the intense photoabsorption of the matrix leads to desorption of entire charged clusters, from which analyte ions are released during desolvation [15]. An alternative model of energy-induced disproportionation (EDIT), proposed by Chang et al., assumes that hydrogen-bonded dimers of the analyte form in the solid phase with the participation of the matrix. After absorption of energy from the laser pulse, energy is transferred to the dimer, leading to the disproportionation and simultaneous formation of a protonated and deprotonated ion. This model provides a good explanation for the observed symmetry in the yield of positive and negative ions for large biomolecules [16]. Another established model, the so-called “happy ion survivors”, assumes the presence of pre-existing but strongly bound matrix and analyte ions in the solid phase. The laser energy does not ionize them per se but breaks the ion–counterion interactions, allowing the release of stable ions into the gas phase. This model explains the presence of intact molecular ions without extensive fragmentation, which distinguishes MALDI from other ionization techniques such as electron impact (EI) or electrospray impact (ESI) [14].

The presence of an organic matrix causes intense signals in the area of *m*/*z* < 1000 Da on the mass spectrum. These signals affect the sensitivity and make it difficult to analyze other compounds due to the presence of a chemical background in the low-mass region [9,17,18]. The solution to this problem may be nanomaterial matrix LDI-MS techniques, in which the organic matrix is replaced with a variety of materials that are applied on the substrate surface. These types of approaches are gaining popularity in the context of analyzing low-molecular-weight compounds due to their ability to generate a low background signal in this *m*/*z* range, which in turn results in a larger number of signals with a high signal-to-noise ratio (S/N). Pavlov and Attygalle demonstrated that LDI, even in a nanomaterial matrix form, is a versatile technique for analyzing a broad spectrum of inorganic compounds, including oxides, salts, and coordination clusters [19].

In the context of SALDI technology, a variety of surfaces are tested for their effectiveness as substrates, with varying degrees of success [20]. By analyzing the elemental composition, most SALDI substrates can be classified into three main categories: carbon-based substrates (such as fullerenes and graphene), semiconductor-based substrates (such as silicon), and metal-based substrates [21,22,23]. Each of these categories is characterized by specific physicochemical properties that can affect the efficiency of the desorption and ionization process and thus the quality and precision of the analytical results. One of the key factors determining the effectiveness of these substrates is the size and morphology of the particles present on their surface, as they directly affect the absorption of laser energy and the efficiency of ion generation. For example, McLean et al. using gold nanoparticles (AuNPs) in the LDI technique showed that AuNPs with a diameter of 10 nm were characterized by a 10-fold higher efficiency in protein detection than their counterparts with a diameter of 2 nm [24]. In turn, Gámez et al., using spherical AuNPs (diameter 30 nm) and AuNP nanorods (diameter 18 mm, length 50 mm) in the LDI technique, noted a 2.8-fold lower laser fluence threshold at 355 nm for spherical AuNPs [25].

LDI methods based on metal nanoparticles, especially noble metals, are becoming increasingly popular in analytical applications. These nanoparticles, composed of metal cations and free surface electrons, exhibit surface plasmon resonance (SPR), enabling strong light absorption and scattering at specific wavelengths. In NALDI, the deposition of metal nanoparticles induces localized surface plasmon resonance (LSPR), which enhances light–matter interactions by concentrating energy into intense local electromagnetic fields. The LSPR response depends strongly on nanoparticle size, shape, and composition, with smaller particles favoring absorption and larger ones favoring scattering [2,4,26]. LSPR involves both intraband (within sp-band) and interband (d–sp transition) excitations, which together shape the material’s optical and plasmonic properties [27,28].

**Figure 2:**
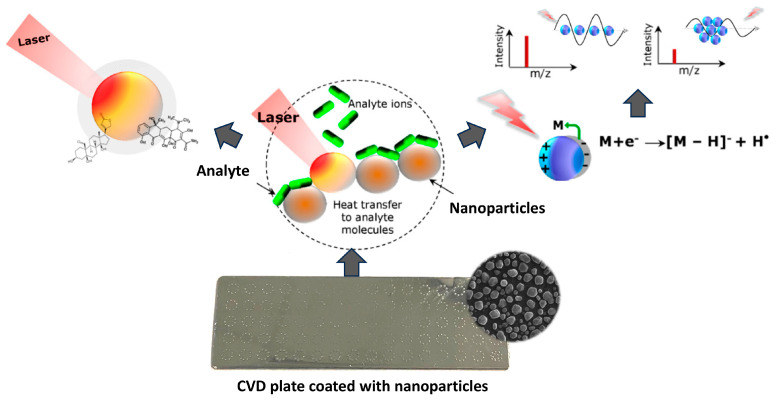
Mechanism of laser desorption/ionization in systems supported by metal nanoparticles.

In NALDI techniques, the desorption and ionization process is also complex and multi-stage, involving both thermal and non-thermal mechanisms. Desorption is usually initiated by the rapid laser-induced heating of the nanostructured substrate (see Figure 2). This effect leads to a local temperature increase, which allows the evaporation of a wide range of analytes [29]. Under very rapid heating conditions, so-called explosive evaporation can also occur, where desorption is driven by density fluctuations in the liquid and not by heat conduction alone [30]. Noble metal nanoparticles covering the substrate surface are effective for thermal desorption due to their strong UV-Vis radiation absorption, low heat capacity, and reduced thermal conductivity [31]. In addition, the desorption process can be supported by non-thermal mechanisms, such as transient destruction or laser-induced surface reorganization, which highlights the complexity of this phenomenon [5]. Desorption is accompanied by the ionization process, which can occur with the participation of high-energy “hot electrons” generated as a result of light absorption by metallic nanoparticles. The transfer of these electrons to molecules adsorbed on the surface can lead to their excitation, dissociation of chemical bonds, or the formation of ions. These effects are crucial for generating an analytical signal in the NALDI technique, where ionization occurs without the participation of a classical, organic matrix [32].

Among d-electrons metals, gold and silver are the two most investigated plasmonic nanomaterials due to their unique optoelectronic properties and ability to excite strong surface plasmon resonances [4,33]. Of the two, silver stands out with its particularly advantageous features—it has the lowest optical losses in the visible and near-infrared range, which translates into high efficiency in spectroscopic techniques, imaging, and detection. Its high electrical conductivity and favorable electronic structure enable intensive local amplification of the electromagnetic field, while strong light scattering and sharp plasmon resonances—especially in the UV-visible range—increase detection sensitivity and analytical resolution [4]. Silver additionally offers better efficiency per unit mass, enables the excitation of multipolar resonances, and is more sensitive to changes in the dielectric environment, making it an exceptionally effective material for advanced optical applications [34]. Moreover, its relatively low price and the good availability of precursors used in vapor deposition techniques favor its use on an industrial scale, e.g., in the production of analytical substrates. Gold, although slightly less effective in terms of plasmonic efficiency, is much more resistant to oxidation and chemical degradation, which makes it a more common choice for applications requiring stability [1].

A number of other metals are also considered for the applications, such as aluminum, copper, palladium, platinum, titanium, cobalt, and nickel. Pangavhane also demonstrated the usefulness of the deposition of chalcogenideglass (As-S-Se) [35]. Although some of them show promising features, their plasmonic properties are usually less favorable than those of silver and gold. Aluminum, for example, enables the excitation of plasmon resonance in the ultraviolet region, which makes it useful in specific applications, but it suffers from higher optical losses and susceptibility to corrosion [36]. Copper, although cheap and having good plasmonic properties, oxidizes quickly, which limits its practical use in stable analytical systems [37]. On the other hand, metals such as palladium, platinum, titanium, and nickel have a more complex electronic structure and higher attenuation, which translates into lower field enhancement and lower efficiency in techniques such as NALDI [38,39].

An important parameter when selecting a metal type for mass spectrometry applications is also its natural isotopic abundance. Metals with many stable isotopes generate ion clusters with complex isotopic spectra, creating so-called “isotopic envelopes”—groups of overlapping peaks that make it difficult to interpret the data unequivocally. In this context, silver, containing only two stable isotopes (^107^Ag and ^109^Ag), is a better alternative. The clear mass deficit between these isotopes and their similar percentage share allows easy identification of Agₙ clusters and their distinction in the mass spectrum [40]. Additionally, the stability and repeatability of Agₙ⁺ cluster signals allow them to be used as internal calibration standards for the precise determination of *m*/*z* values in the range of up to 1000 Da; as literature reports show, such a solution allows for the simultaneous calibration and biodetection of small molecules [41,42]. It is worth emphasizing, however, that the presence of multiple cluster forms, such as [M + Ag_2_]^+^ or [M + Ag_3_]^+^, is also possible in the mass spectrum, which increases the number of signals and may complicate the interpretation of results, especially in the case of samples with a complex composition. On the other hand, the formation of specific [M + Ag_n_]^+^ adducts is strictly determined by the chemical nature of the analyte, which means that it can be an additional identification parameter supporting the distinction of compounds with a similar mass and even enable inference about the presence of specific functional groups or molecular structures.

## 3. Chemical Vapor Deposition as a Method for Synthesizing Silver Nanoparticles

There are two primary groups of methods that are used in the fabrication of metal nanomaterials: top-down methods and bottom-up methods [43,44]. The top-down approach, often referred to as “building from top to bottom”, involves reducing the dimensions of fabricated elements through specialized equipment or etching techniques. This group of methods is based on advanced lithographic techniques, including nanoprint lithography, soft lithography, scanning tunneling microscope-based methods, and 3D direct laser writing [1]. The second group, the bottom-up approach, focuses on constructing materials from the ground up, atom by atom or particle by particle. This category includes methods such as self-assembly, direct doping with nanoparticles, vapor-phase deposition techniques, sol-gel processing, and colloidal synthesis [44,45]. It is generally easier to construct nanoparticles atom by atom or molecule by molecule, as this approach offers better control over their structural properties, leading to more uniform, high-purity, and precisely tailored products.

An interesting group of methods for the synthesis of metallic nanoparticles, especially in terms of applications in advanced analytical techniques such as NALDI, are methods based on chemical vapor deposition. The most important techniques of this type include chemical vapor deposition (CVD), atomic layer deposition (ALD), and physical vapor deposition (PVD) [46,47,48]. In CVD, the deposition of material occurs through chemical reactions on the substrate surface, which allows for control of the morphology of nanoparticles and their optical and plasmonic properties. ALD, a specialized type of CVD, uses sequential, self-limiting surface reactions, thanks to which it is possible to obtain nanoparticles of exceptionally uniform size and distribution, which is important for demanding applications such as the production of precise substrates for NALDI. However, this method is more complex, time-consuming, and expensive. PVD, on the other hand, relies on physical processes such as evaporation or sputtering, offering simplicity and ease of scaling, but provides less precise control over the size and morphology of nanoparticles, which may limit its use in advanced analyses requiring high homogeneity [49,50,51,52]. Due to the balance among precise control, scalability, and cost-effectiveness, CVD seems to be the most reasonable choice for the synthesis of metallic nanoparticles for NALDI applications, enabling the production of materials with tailored properties while ensuring the efficiency of production at a larger scale.

In chemical vapor deposition, chemical reactions are driven by thermal energy to deposit material onto a substrate, but alternative energy sources can also be employed to activate these reactions, resulting in various CVD modalities. For instance, plasma-enhanced CVD (PECVD) leverages plasma to accelerate reaction kinetics, flame-assisted CVD (FACVD) uses flame energy for precursor decomposition, and laser-activated CVD (LCVD) employs laser irradiation for localized heating and reaction facilitation. CVD processes can operate under atmospheric pressure (APCVD) or reduced pressure (LPCVD), with each mode offering distinct advantages tailored to specific applications [50].

**Figure 3:**
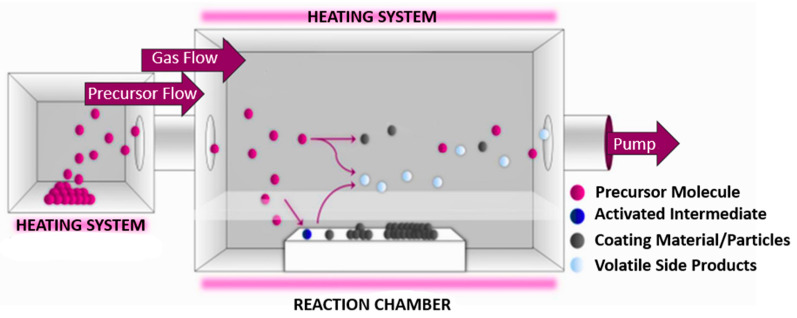
Schematic representation of the CVD process.

The typical CVD process comprises several critical stages that are essential for the successful deposition of nanomaterials. Figure 3 illustrates the CVD process [43]. Initially, the substrate undergoes preparation and activation to optimize surface properties for nanomaterial adhesion. This step may include cleaning, etching, or applying a specific coating. Subsequently, the precursor is introduced into the reactor chamber and vaporized through sublimation or evaporation, ensuring its transformation into a gaseous phase suitable for transport. The precursor vapors, often mixed with a carrier gas, are then transported to the deposition zone, where surface reactions occur under controlled conditions. These reactions involve the formation of reactive intermediates, which are adsorbed onto the substrate surface. At the gas–solid interface, chemical reactions proceed to initiate nucleation, embryo growth, and ultimately the formation of a continuous material layer. Concurrently, reaction by-products are desorbed and removed from the reactor to maintain process integrity and prevent contamination [52]. The deposition process itself requires the prior heating of the reactor to the appropriate temperature, which—depending on the heating system and experimental assumptions—may take from 30 min to an hour. The proper deposition time of metal nanoparticles such as silver depends on the type of precursor used and the process conditions, usually ranging from several dozen minutes to two hours. The ability of CVD to accommodate diverse energy sources and pressure conditions, coupled with its scalability, underscores its robustness and industrial relevance. This versatility positions CVD as a powerful tool for producing high-quality metal nanoparticles for a broad range of applications.

### 3.1. Silver Precursors

In CVD techniques used for the synthesis of silver nanoparticles, the key aspect determining the efficiency of the process and the quality of the obtained layers is the proper selection of the precursor. The ideal precursor should be characterized by appropriate volatility, moderate thermal stability (enabling decomposition in the appropriate temperature range), and high chemical purity, which has a direct impact on the purity of the deposited nanoparticles. Although many chemical compounds can meet basic physicochemical requirements, in practice many of them pose significant challenges—too high thermal stability, low flash points, or too high reactivity may limit their use in conditions typical for CVD processes [53,54]. Generally, precursors can be divided into inorganic, organometallic, and metal–organic complexes. An overview of important precursors for silver nanoparticles is presented in Table 1.

Among the inorganic silver precursors, silver nitrate (AgNO_3_) is widely used and is mainly used in APCVD and FACVD. Despite its availability and ease of use, the high evaporation temperature of silver nitrate limits its usefulness in some CVD variants, especially where lower process temperatures or sensitive substrates are required. Silver halides, such as silver(I) fluoride (AgF), also require high temperatures, which makes them less flexible in the context of technological applications [54].

Much more promising are organometallic precursors and organometallic complexes, which, due to the presence of organic ligands, allow for lower decomposition temperatures and increased volatility. In particular, silver(I) complexes with β-diketonates and carboxylates—both fluorinated and non-fluorinated—are being studied. These variants are suitable for techniques such as MOCVD, AACVD, or PECVD, where control over the growth parameters and structure of layers is required. Complexes with ligands such as trimethylphosphine (PMe_3_), triethylphosphine (PEt_3_), vinyltriethoxysilane (VTES), bis(trimethylsilyl)acetylene (BTMSA), and bis(trimethylsilyl)ethyne (BTMSE) show better thermal stability and lower decomposition temperatures than their counterparts without these ligands. This enables the efficient and controlled deposition of silver nanoparticles of uniform size and shape [48,55,56,57].

Some carboxylates, such as silver acetate (AgOAc) or silver(I) trifluoroacetate, despite their limited volatility, can be effectively used in techniques requiring a local and intensive source of energy, e.g., in laser-activated CVD (LCVD), where they enable the selective and precise deposition of metal layers. During the process, these compounds decompose thermally, releasing silver atoms, which condense on the substrate in the form of a nanostructured layer [57].

Among the more modern precursors, silver(I) pentafluoropropionate draws special attention, which, thanks to its high volatility and low decomposition temperature under LPCVD (CVD under reduced pressure) conditions, enables the rapid deposition of silver layers. Continuous, smooth layers can be obtained after 30 min of deposition. This compound is distinguished not only by its chemical parameters but also by its simplicity of synthesis and relatively low costs. Its trihydrate form ([Ag_5_(OOCC_2_F_2_)_5_(H_2_O)_3_]) shows additional chemical and photochemical stability, which facilitates its storage and use under standard conditions [54,56,58].

Another example of a promising precursor is silver(I)-2-[2-(2-methoxyethoxy)ethoxy] acetate, which is characterized by good solubility in common polar solvents and stability in air. Its structure allows for the deposition of thin silver layers of a controlled thickness (from 5 to 30 min of the process) as well as obtaining multilayer systems [59].

The properties of the obtained layers—such as morphology, purity, homogeneity, or density of nanoparticles—depend directly on the thermal decomposition mechanism of a given precursor, the type of ligand, and the process conditions (pressure, temperature, composition of the reaction atmosphere). Therefore, the careful selection and engineering of precursors play a fundamental role in the optimization of CVD processes aimed at the synthesis of silver nanoparticles for highly specialized applications, such as matrices for NALDI-MS mass spectrometry [54].

### 3.2. Oxidation of Silver

Studies on multinuclear silver precursors, mostly those that contain polyfluorinated or phosphine ligands, demonstrated advancements in controlling the oxidation of silver layers and enhancing their deposition properties. For instance, silver complexes with perfluorinated carboxylates exhibited high thermal stability and uniform layer formation due to the strong Ag–ligand interactions [57,60]. Similarly, polynuclear silver(I) complexes with diphosphine ligands enabled controlled structural diversity, which is crucial for forming dense, smooth layers with minimal defects during chemical vapor deposition [61]. Complexes with tri-n-butylphosphine exhibited low susceptibility to decomposition during evaporation, resulting in smooth surface layers with high electrical conductivity [62]. Sibińska et al. obtained a uniform layer of silver nanoparticles (AgNPs) on a steel substrate via CVD using the precursor [Ag_5_(O_2_CC_2_F_5_)_5_(H_2_O)_3_]. Although oxygen and carbon from atmospheric O_2_ and CO_2_ were adsorbed onto the surface, promoting slight oxidation, the silver oxide content remained low—around 1% after one month [42].

### 3.3. Functional Applications and Characterization Techniques of Silver Nanoparticles

Silver nanoparticles synthesized by CVD are useful in implantology and biomedicine. Studies showed their cytotoxicity toward various cell models [63,64]. Radtke et al. highlighted the microbiocidal and biocompatible properties of AgNPs on titanium- coated alloys [65]. Another study examined the deposition of AgNPs onto titanium modified with TiO_2_ nanotubes, analyzing surface roughness, wettability, mechanical properties, and silver ion release. Also, Piszczek and Radtke provided a comprehensive review of the fabrication, properties, and biomedical applications of AgNPs produced via CVD and atomic layer deposition techniques [48]. These studies collectively highlight the versatile properties of AgNPs synthesized through CVD, ranging from enhanced SERS activity and optoelectronic functionalities to antimicrobial and biocompatible characteristics, making them valuable across multiple scientific and technological domains.

In nanotechnology and materials engineering, silver nanoparticles obtained by CVD are used to modify carbon structures, such as multi-walled carbon nanotubes (MWCNTs). Their decoration with AgNP leads to the creation of composites with improved mechanical properties, electrical conductivity, and thermal stability. The uniform dispersion of silver in the nanotube structure also affects the enhancement of plasmonic effects, which broadens the range of applications of such materials, including in optical devices, catalysis, and energy storage systems [66].

**Figure 4:**
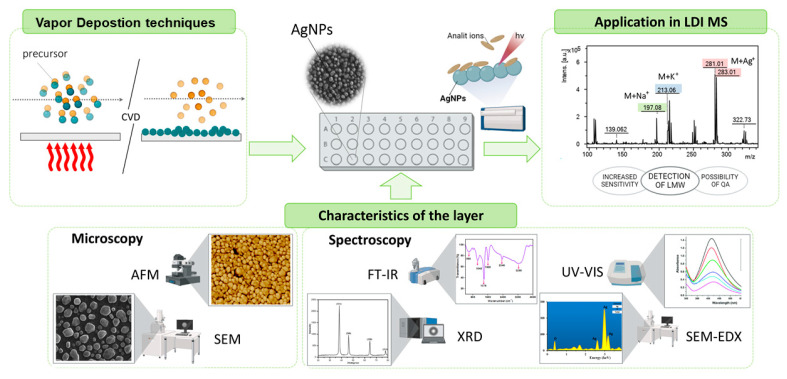
Scheme of CVD synthesis, characterization of the AgNP layer, and its application in the LDI technique.

At the same time, CVD-produced AgNPs exhibit unique optoelectronic features that are crucial in advanced analytical techniques. Silver nanoparticles are used in surface-enhanced Raman scattering applications, where the localized electromagnetic fields around AgNPs significantly amplify the Raman signals of adjacent molecules, thereby enabling highly sensitive and accurate detection [67]. In sensing, AgNPs can enhance the sensitivity of sensors, enabling the detection of minute quantities of analytes. In catalysis, their large surface area and unique electronic properties improve the efficiency and selectivity of catalytic reactions. The use of AgNPs in NALDI-TOF MS enhances ionization efficiency and reduces matrix effects, resulting in the improved sensitivity and accuracy of mass spectrometric analyses [52,67] (see Figure 4).

The characterization of thin layers, including silver nanoparticles, necessitates the use of advanced analytical techniques capable of precisely evaluating their thickness, chemical composition, structural properties, and surface characteristics. Primarily, microscopic and spectroscopic techniques are employed for this purpose. Microscopic techniques, including Scanning Electron Microscopy (SEM, High Resolution-SEM) and (Atomic Force Microscope) AFM, are utilized to evaluate the topography, thickness, and roughness of the layer as well as to determine the shape, size, and distribution of nanoparticles comprising the layer. In turn, valuable information about the properties and composition of nanoparticles can be obtained through spectroscopic methods, which analyze various interactions of electromagnetic radiation with matter, such as absorption, emission, and scattering. These techniques provide critical insights into the characteristics of thin layers and nanoparticles, including energy dispersive spectroscopy (EDS) for elemental composition analysis; X-ray diffraction (XRD) for determining crystal structure, phase identification, and lattice parameters; X-ray photoelectron spectroscopy (XPS) for analyzing electronic structure, elemental composition, chemical bonding, and oxidation states; Fourier transform infrared spectroscopy (FT-IR) for investigating chemical compositions, functional groups, and chemical bonding; Raman spectroscopy for investigating vibrational modes, molecular interactions, and the structural properties of nanoparticles UV-Vis spectroscopy (UV-VIS) for characterizing absorption spectra and functional group transitions; and ultraviolet photoelectron spectroscopy (UPS) for examining electronic band structure, chemical composition, and surface sensitivity [47,68].

## 4. Application of LDI-MS Techniques

Laser desorption/ionization techniques, such MALDI and SALDI, offer plenty of advantages over other analytical methods in various contexts due to their unique characteristics. Their high sensitivity enables the detection of trace compounds in quantities as small as the femtomole to attomole range, making them ideal for applications in biological, environmental, and chemical analyses. LDI techniques also stand out for their minimal sample preparation requirements. These methods are versatile, capable of analyzing diverse compounds ranging from small molecules to biomolecules like proteins and nucleic acids, and are relatively non-destructive, allowing for subsequent analyses if needed. LDI techniques are renowned for their speed, with analysis times ranging from seconds to minutes, and their suitability for high-throughput applications, especially when automated [29]. The analysis time in the LDI technique using AgNPs remains comparable to the time typical for methods based on traditional organic matrices. However, the key advantage of this approach is the simplification of the sample preparation procedure—the analyte can be directly applied to the plate surface without the need for co-crystallization with the matrix, which simplifies and speeds up the entire process.

LDI techniques are widely applied in various analytical fields, with MALDI being the most commonly utilized method. This technique is extensively used in science and industry, particularly in microbiology for rapid bacterial and fungal identification as well as antibiotic resistance testing [69]. It has clinical applications, such as analyzing microorganisms in diabetic foot wounds [70] and is also employed in medicine to identify disease biomarkers [71], analyze drugs [72,73], and monitor therapies [17]. MALDI enables the study of biological samples like serum, urine, and cerebrospinal fluid and supports drug research by analyzing drug–protein interactions, pharmacokinetics, and metabolites [74]. In the food and agrochemical industries, it is used for contaminant detection, quality control, and chemical composition analysis [75]. Nevertheless, the analysis of low-molecular-weight compounds, such as lipids, carbohydrates, peptides, and drugs, remains a significant challenge.

LDI techniques can also be used as analytical tools in the analysis of petrochemical compounds. The ability of MALDI to quickly identify and characterize impurities in petrochemical products allows for improve the monitoring of product quality and production processes, analyzing complex hydrocarbon mixtures, detecting contaminants, and ensuring fuel quality [76,77,78,79]. MALDI has been extensively utilized for the characterization of asphaltenes, high-molecular-weight components in crude oil that impact refining processes. Studies demonstrated that MALDI can effectively analyze asphaltenes without extensive sample preparation. It offers the rapid assessment of their molecular weight distribution and compositional information [80]. This capability is critical for optimizing refining processes and ensuring the efficient conversion of crude oil into valuable products. MALDI-MS is also used in identifying various compounds, such as aromatic hydrocarbons and nitrogen-containing molecules, important for understanding the physicochemical properties of crude oil [81]. A noteworthy application of MALDI was in the study conducted by researchers from Austria. They investigated the chemical and structural changes in oil and its additives during exploitation using MALDI. The analysis, performed in both positive and negative ion modes, employed matrices like CHCA and DHT (dithranol). The study revealed that the DHT matrix provided better results in the negative ion mode, while HCCA was more effective in the positive ion mode [82]. This differentiation in matrix performance highlights the need for careful matrix selection to optimize analysis outcomes. Recent studies also highlighted the utility of LDI in petrochemical justification, including the elimination of biosurfactants consumed by hydrocarbon-degrading bacteria, which supports the bioremediation of oil-contaminated environments [83].

Despite its advantages, MALDI-MS faces several limitations, particularly in the analysis of low-molecular-weight compounds, which are prevalent in samples like petroleum. The technique’s reliance on a suitable matrix can sometimes interfere with the analysis of hydrocarbons by introducing additional peaks and complicating spectra interpretation. This matrix effect can obscure the analyte signals, making it challenging to obtain clear and accurate results. Additionally, the ionization efficiency for non-polar hydrocarbons is relatively low, limiting the technique’s applicability for certain types of analyses. Addressing these limitations requires the development of improved matrices or alternative ionization methods to enhance LDI performance [9]. This approach can be extended to NALDI-TOF MS for the rapid screening and analysis of contaminants in diesel and other fuels, ensuring high-quality standards and compliance with environmental regulations. The use of nanoparticles synthesized by the CVD method offers additional possibilities, enabling greater control over their size, shape, and uniformity, which can further enhance the sensitivity and precision of the analysis.

While the applications of LDI-MS in the petrochemical industry show significant promise, several challenges remain. The variability in diesel composition across different regions and the presence of diverse additives can complicate the analysis. Moreover, the high sensitivity of LDI-MS techniques, while advantageous, can sometimes lead to the detection of background noise, necessitating further refinement in sample preparation and data analysis methods.

### Role of Silver Nanoparticles in LDI Techniques

Metal nanoparticles are increasingly being recognized in the scientific literature as an alternative nanomaterial matrix that effectively facilitates the ionization of diverse types of samples. The most commonly used methods of synthesizing nanoparticles (e.g., silver, gold) for LDI are based on the chemical reduction in salts [84,85] or electrochemical growth on the cathode surface [86]. For instance, Arendowski et al. demonstrated the use of electrochemically deposited monoisotopic cationic ^109^Ag nanoparticles in LDI-MS for detecting and quantifying amino acids, highlighting the method’s high sensitivity and rapid measurement capabilities [87]. A study by Prysiazhnyi et al. emphasized the utility of gas-aggregated Ag nanoparticles in detecting small molecules (amino acids, sugars and derivatives, vitamins, antibiotics, lipids, and PAH hydrocarbons), highlighting their potential to replace traditional matrices in LDI [88]. Furthermore, Kołodziej et al. presented an innovative approach using infrared pulsed fiber laser-produced AgNPs for LDI-MS of carboxylic acids [89]. This method employed a pulsed fiber laser (PFL) with a galvo-scanner for laser ablation synthesis in solution (LASiS) to produce high-resolution LDI-MS and mass spectrometry imaging (MSI) of carboxylic acids. The study quantified six test acids under varying concentrations and tested the method on spiked human blood serum samples, demonstrating its potential for high-sensitivity analysis [89]. Silver nanoparticles can be also used for the selective ionization of specific groups of compounds, such as olefins, estrogens, and antibiotics. AgNPs enable the efficient ionization of olefinic compounds (e.g., cholesterol, carotenoids) even without sample cleanup. They are also effective for detecting estrogens and sulfur-containing drugs because of van der Waals and electrostatic interactions [90]. However, these methods require further refinement in terms of the amount of silver deposition, nanometric layer control, and reproducibility. Moreover, the size of the AgNPs plays a critical role in their performance in LDI-MS.

Achieving precise control over these parameters is critical for ensuring the reliability and efficiency of LDI-MS analyses. Here, CVD offers a robust solution by enabling meticulous control over the deposition conditions, ensuring consistent and high-quality nanolayer formation on various substrates [5,91]. Sagandykova et al. investigated the fabrication of silver nanostructured substrates via CVD and demonstrated their effectiveness in enhancing laser desorption/ionization mass spectrometry sensitivity for various low-molecular-weight biomolecules. The study revealed that the controlled deposition of silver nanoparticles on substrates, specifically NALDI targets on stainless steel 316L, significantly improved signal intensity in mass spectrometric analysis, facilitating the detection of minute quantities of biomolecules [92]. The versatility of CVD in handling diverse materials and the ability to fine-tune critical parameters such as precursor concentration, growth temperature, and deposition rate renders it a robust and adaptable method for synthesizing high-quality AgNPs’ nanoparticles. The produced NALDI plates with CVD-derived AgNPs were also utilized in the metabolic profiling of bacterial extracts obtained using the Bligh and Dyer method. These substrates proved effective in differentiating between Gram-positive (G+) and Gram-negative (G−) species and in clustering analysis across all eight bacterial species studied [93]. Furthermore, Sibińska et. al. presented a study on the use of AgNPs, synthesized via CVD, to improve the performance of laser desorption/ionization mass spectrometry. The AgNPs produced were uniformly distributed, offering the more sensitive detection of LMW biomolecules, such as amino acids, lipids, and metabolites. This technique enhances ionization efficiency while reducing background noise, critical for improving signal clarity in LDI applications [42]. Also, Maślak et al. highlighted the application of CVD-produced AgNPs in bacterial lipidome analysis, showing their potential in antibiotic susceptibility testing. The study showed the low background noise in nanostructure-assisted LDI compared to matrix-assisted LDI, which enhances the detection sensitivity and accuracy. This reduced background noise is important for clear signal detection, making AgNPs a good choice for LDI applications [94].

## 5. Conclusions

In NALDI MS, AgNPs improve ionization efficiency, enhancing the sensitivity and precision of mass spectrometric analyses. This is important for detecting low-molecular-weight analytes and targets fabrication and complex biological samples, making NALDI an invaluable tool in analytical chemistry and environmental monitoring. The method of nanoparticle deposition onto the surface appears to be a critical determinant in achieving optimal performance. The AgNPs produced by CVD are highly versatile and effective in a wide range of applications. The CVD method offers precise control over nanoparticle structure and properties. It is achieved through the careful manipulation of reactor design, precursor selection, and deposition conditions. This precision makes CVD-synthesized AgNPs valuable in fields such as laser desorption/ionization mass spectrometry.

Moreover, LDI-MS techniques employing AgNPs are gaining attention in the petrochemical industry. These methods enable the detailed analysis of petrochemical matrices, such as diesel, by accurately identifying hydrocarbons and contaminants. Such analyses are essential for ensuring fuel quality, maintaining engine performance, and meeting stringent environmental regulations. The ability to monitor fuel composition and impurities swiftly and accurately helps optimize fuel performance and reduce environmental impact. The integration of AgNPs synthesized by CVD into advanced analytical techniques underscores the synergy between material science and practical applications. The ongoing development and refinement of these technologies promise further enhancements in efficiency, sensitivity, and accuracy, driving innovation across both scientific research and industrial practices.

Future research should focus on enhancing the robustness of LDI-MS techniques for routine petrochemical analysis. This includes developing more efficient sample preparation protocols, improving matrix and nanoparticle substrates, and integrating advanced data analysis tools. Additionally, the environmental impact of using nanoparticles and other materials in LDI-MS should be carefully considered, promoting sustainable practices in analytical chemistry.

## Figures and Tables

**Table 1:** Comprehensive overview of silver-based precursors for chemical vapor deposition (CVD) applications.

Precursor	Type	Key Characteristics	Applications in CVD	Refs.
Silver nitrate (AgNO_3_)	Inorganic	High decomposition temperature, limited volatility	Atmospheric pressure CVD, flame-assisted CVD	[54]
Silver(I) fluoride (AgF)	Inorganic	High temperature required for evaporation and reaction	High-temperature CVD	[54]
Silver(I) β-diketonates	Organometallic	Volatile, thermally stable	MOCVD, AACVD, plasma-enhanced CVD	[55,56]
Silver(I) carboxylates	Metal–organic complex	Low volatility, thermally stable	Laser-activated CVD	[57]
Silver(I) acetate (AgOAc)	Metal–organic complex	Low volatility	Laser-activated CVD	[57]
Silver(I) trifluoroacetate	Metal–organic complex	Low volatility, fluorinated	Specialized techniques	[54,57]
Silver(I) pentafluoropropionate (Ag_5_(OOCC_2_F_2_))	Metal–organic complex	High volatility, low decomposition temperature	Low-pressure CVD	[54,56,58]
Silver(I) pentafluoropropionate trihydrate	Metal–organic complex	Enhanced stability at ambient conditions	Low-pressure CVD	[54,55,58]
Silver(I)-2-[2-(2-methoxyethoxy)ethoxy]acetate	Metal–organic complex	Air-stable, polar solvent soluble, low-cost synthesis	Thin silver layers, multilayer systems	[59]

## Data Availability

The data presented in this study are available in this article.
